# High‐Quality Annotated ZZ Male Genome Assembly of *Eulimnadia texana*

**DOI:** 10.1002/ece3.74547

**Published:** 2026-10-23

**Authors:** Chathumadavi Ediriweera, Stephen C. Weeks

**Affiliations:** ^1^ Department of Biology The University of Akron Akron Ohio USA

## Abstract

In many animals, sexual differences are predominantly determined by sex chromosomes and therefore understanding the underlying evolutionary mechanisms that affect these chromosomes is crucial. Most studies on sex chromosome evolution have employed model organisms with ancient sex chromosome systems, which have already undergone evolutionary degradation thereby hindering the detection of early evolutionary processes. Androdioecious crustaceans in the genus *Eulimnadia* have a unique sex‐determining system that allows examination of evolutionary models of early sex chromosome evolution. In typical ZW sex chromosome systems, the Z is usually homogametic (i.e., ZZ) and the W is heterogametic (i.e., ZW), the latter being prone to long‐term degeneration due to a lack of recombination. In *Eulimnadia texana* , both Z and W chromosomes are found in homogametic form (i.e., ZZ and WW) retarding the buildup of deleterious recessive alleles thereby keeping these chromosomes in the “proto‐sex chromosome” stage of evolutionary development. Therefore, the *E. texana* system allows exploration of the early stages of W and Z sex chromosome development. Although there is a high‐quality assembly of WW hermaphrodites, there is no complementary high‐quality male (ZZ) genome assembly yet for *E. texana* . In the current study, we generate PacBio HiFi sequencing data from a ZZ male, construct a contamination‐filtered de novo genome assembly, and then scaffold this assembly against the WW hermaphrodite reference. The de novo ZZ contig assembly spans ~113 Mb with an N50 of 3.5 Mb and 98.1% complete Arthropoda BUSCO genes, and scaffolding against the WW reference yields a chromosome‐scale ZZ genome (12 scaffolds, total length ~113 Mb, scaffold N50 18 Mb, 0.18% gaps). Together with its gene annotation, this scaffold‐level ZZ genome complements the WW assembly and enables future comparative analyses of the Z and W proto‐sex chromosomes.

## Graphical abstract

Androdioecious crustaceans in the genus *Eulimnadia* have a unique sex‐determining system that allows examination of evolutionary models of early sex chromosome evolution. Although there is a high‐quality assembly of WW hermaphrodites, there is no complementary high‐quality male (ZZ) genome assembly yet for *E. texana* . In the current study, we present a comprehensive ZZ male genome assembly for *E. texana* that allows a comparison of the Z to the W genomes, which is useful for testing predictions of sex chromosome evolutionary theory.

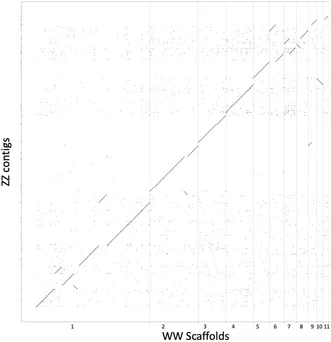

## 1 Introduction

In many animal species, sex is determined genetically, often through sex chromosomes (Bachtrog et al. 2014). There are two common sex chromosomal systems: (1) The more common sex chromosome system is the XY system (male heterogamety), found in mammals (including humans and mice), some insects (e.g., *Drosophila*, mosquitoes, *Musca domestica* and *Ceratitis capitata* ), medaka fish, and nematode worms, where males carry XY chromosomes and females XX (Nanda et al. 2002; Arnold 2009; Gempe and Beye 2011; Bachtrog et al. 2014; Biedler and Tu 2016; Wang et al. 2022); and (2) The less common arrangement is the ZW system (female heterogamety), common in birds, reptiles, lepidoptera, some fish species and many crustaceans where females carry ZW chromosomes (female heterogamety), and males are ZZ (Bull 1983; Ezaz et al. 2006; Bachtrog et al. 2014; Irwin 2018; Ye et al. 2023). Sex chromosomes are the only pair of chromosomes that are not represented equally in males and females. Sex‐limited chromosomes commonly have limited to no crossing over and they have one sex chromosome (Y or W) that is “sex‐limited” (i.e., always found as XY or ZW, depending on species) (Charlesworth et al. 2005; B. Charlesworth 1978; Bachtrog et al. 2014; Rifkin et al. 2021). These characteristics have led to the degeneration of the Y (or W) in many species, including humans (Bull 1985; Rice 1996; Charlesworth and Charlesworth 2000; Hughes and Page 2015).

One of the hypotheses for the degeneration of the Y (or W) chromosome is that the lack of recombination in the sex‐limited chromosome drives sex chromosomes to degrade (B. Charlesworth 1978; Bull 1985; Rice 1996; Charlesworth and Charlesworth 2000; Charlesworth et al. 2005). When recombination is suppressed, deleterious alleles will be harder to eliminate; instead, they can be linked to “neutral” or even “beneficial” genes and thus be harder to eliminate from the non‐recombining chromosome (Rice 1996; Charlesworth and Charlesworth 2000). Additionally, the accumulation of transposable elements (TEs) will take place on the sex‐limited (Y or W) chromosome for similar linkage reasons, potentially causing damage to gene function and chromosomal rearrangement (B. Charlesworth 1978; Rice 1996; Charlesworth and Charlesworth 2000; Medstrand et al. 2005). Consequently, accumulation of these deleterious alleles and TEs in the non‐recombining region of the sex‐limited chromosome should initiate the process of overall chromosomal degradation (Rice 1996; Charlesworth and Charlesworth 2000). In addition to the linkage issues, the permanent heterozygosity of the Y (or W) chromosome can shelter selection against recessive deleterious mutations because of masking by wild type genes on the X (or Z) (Rice 1996; Charlesworth and Charlesworth 2000).

When testing hypotheses of sex chromosome degradation, the major challenge is that most studies of sex chromosome evolution in animals have been conducted using model organisms, which are commonly dioecious with evolutionarily old, highly diverged sex chromosomes (Bachtrog et al. 2011; Bachtrog 2013; Abbott et al. 2017). To understand the underlying evolutionary mechanisms driving the transition from morphologically similar (homomorphic) to differentiated (heteromorphic) sex chromosomes, it is important to study systems with younger sex chromosomes (i.e., “proto‐sex chromosomes”) which are at earlier evolutionary stages (Bachtrog 2013).

*Eulimnadia texana* is an excellent candidate to serve as such a study system, as these “clam shrimp” appear to have proto‐sex chromosomes (Nakamura 2009; Lang et al. 2024; Ediriweera and Weeks 2024, 2026). These clam shrimps have an androdiecious (males and hermaphrodites) breeding system, which is rare in both plants and animals (Lloyd 1975; B. Charlesworth 1978, 1984; Pannell 2002; Weeks, Benvenuto, and Reed 2006; Weeks, Sanderson, et al. 2006; Weeks et al. 2008; Weeks 2012). *Eulimnadia texana* is an androdioecious branchiopod crustacean which has three common mating types formed by combining two proto‐sex chromosomes: Z and W (Sassaman and Weeks 1993; Weeks et al. 2010). Males are homozygous for the Z male chromosome (ZZ), while hermaphrodites are ZW or WW, the latter being highly unusual in most animal species (Sassaman and Weeks 1993). Because *E. texana* has both the Z and W chromosomes present in homogametic form, deleterious recessive alleles on both sex chromosomes can be purged via expression in homogametic males (ZZ) and hermaphrodites (WW). This unique feature allows assessment of the effect of “sheltering” the W chromosome in permanent heterozygosity for the degeneration of the W sex chromosome (D. Charlesworth 1984; Pannell 2002). Therefore *E. texana* is an ideal species for exploring the earlier stages of sex chromosome evolution.

To understand the potential of this system, it is important to characterize the nuclear genomic assemblies of both male and hermaphrodite *E. texana* . The WW hermaphrodite genome is already sequenced and assembled (Baldwin‐Brown et al. 2018). The genome size of the WW was found to be ~120 Mb with 108 scaffolds; additionally, 30% of the genome harbored repetitive elements (Baldwin‐Brown et al. 2018). The current study augments the WW genomic assembly by reporting the full sequence of the ZZ male genome, followed by de novo assembly and annotation to explore the DNA composition in ZZ males. Here, we first generate PacBio HiFi sequencing data from a ZZ male and perform a contamination‐filtered de novo contig‐level assembly (97 contigs, total length ~113 Mb, contig N50 3.5 Mb) with 98.1% complete BUSCO genes and then used the contig‐level assembly against the WW reference to assemble a scaffolded ZZ genome with chromosome‐scale scaffolds (12 scaffolds, scaffold N50 18 Mb).

## 2 Materials and Methods

### 2.1 Shrimp Rearing

Male shrimp were reared using a soil sample containing resting eggs of *Eulimnadia texana* . This soil sample was obtained from a 10th generation laboratory experiment where these males were derived from a line (originally sampled from Jornada, New Mexico) that experienced eight generations of selfing in WW hermaphrodites (Weeks 2004), followed by a cross to non‐inbred ZZ males and then two additional generations of selfing and/or random mating (Calabrese and Weeks 2018); because the presence of males does not ensure offspring are produced via outcrossing (Weeks et al. 2004), mixtures of males and hermaphrodites result in various proportions of selfed and outcrossed offspring. We employed the same techniques and rearing conditions outlined in Calabrese and Weeks (2018). By Days 9 or 10, sexually mature shrimp were removed from the tank, observed under a microscope to identify males, and then the males were flash‐frozen for DNA extraction.

### 2.2 DNA Extraction

Whole, sexually matured male shrimps were kept in a −80°C freezer until DNA extraction was performed at DNA Services, University of Illinois at Urbana‐Champaign, IL, USA. The genomic DNA (gDNA) of each male individual was extracted with the Qiagen Magattract kit (Qiagen, Germantown, MD, USA) using the HMW DNA protocol (https://www.qiagen.com/us/resources/resourcedetail?id=3e331b6c‐0742‐483b‐b74f‐a7be2d4c1116&lang=en) with the following modifications: (1) samples were mixed by gentle inversion or pipette mixing with wide‐bore tips instead of shaking; (2) tissue was ground to a fine powder in liquid nitrogen; (3) during lysis, samples were incubated at 56°C for 1 h and then at room temperature overnight; (4) the water‐rinse steps were omitted to maximize DNA recovery; and (5) elution was performed at 37°C to increase DNA yield. To improve DNA purity for ultra‐low‐input library preparation, the extracted gDNA was passed through a Zymo OneStep PCR Inhibitor Removal column, which is designed to remove common PCR inhibitors (e.g., residual organic compounds and salts) that may co‐purify with DNA from whole‐organism extractions. This step was intended to improve downstream amplification efficiency, not to remove non‐target genomic DNA. Among these individuals, the single sample with the highest‐quality gDNA was chosen for library preparation and sequencing.

### 2.3 Library Preparation and Sequencing

Genomic DNA from a single ZZ male was used for library preparation and PacBio HiFi sequencing, which were conducted at DNA Services, University of Illinois at Urbana‐Champaign, IL, USA. The gDNA was PCR‐amplified with the Ultra‐Low Input kit from PacBio using the “AAGCAGTGGTATCAACGCAGAGTACT” adaptor, targeting an average insert size of ~10 kb as recommended for Ultra‐Low HiFi libraries. Amplified gDNAs were converted to a SMRTBell library with the SMRTBell Express Template Prep kit 2.0 (Pacific Biosciences) and sequenced on one SMRT cell 8M on a PacBio Sequel II using the Circular Consensus Sequencing (CCS) mode with a 30hs movie time. CCS analysis was done using SMRTLink V11.1 (ccs ‐‐min‐passes 3 ‐‐min‐rq 0.999), trimming the DNA_Sample_Amplification_Adapter (AAGCAGTGGTATCAACGCAGAGTACT), marking PCR duplicates, and removing primer sequences, which yielded adapter‐free, deduplicated HiFi reads. Read quality was assessed with FastQC (Andrews 2010).

### 2.4 Genome Size Estimation

We estimated genome size, heterozygosity, and repeat content from PacBio HiFi reads using Jellyfish v2.2.7 to generate *k*‐mer counts and GenomeScope 2.0 to fit *k*‐mer models (ploidy = 2, *k* = 21; the standard GenomeScope default for genomes of this size), providing a rough, reference‐free profile of genome characteristics.

### 2.5 Overview of Assembly Workflow

In summary, our assembly workflow proceeded in three stages. First, we generated a preliminary de novo ZZ contig assembly and used BlobToolKit to remove non‐clam shrimp contigs, then reassembled only remapped reads to produce a contamination‐filtered de novo ZZ contig set. Second, we applied the same BlobToolKit filtering and TGS‐GapCloser gap filling to the published WW hermaphrodite genome to obtain a curated reference. Third, we used RagTag to scaffold the filtered ZZ contigs onto the curated WW scaffold‐level reference genome, yielding a scaffold‐level ZZ genome assembly.

### 2.6 De Novo Genome Assembly

We generated a preliminary contig‐level de novo ZZ assembly following the VGP pipeline (Rhie et al. 2021) on the Galaxy platform (https://usegalaxy.org/), using HiFiasm (Galaxy v0.16.1 + galaxy4) (Cheng et al. 2021) for contig‐level assembly and duplicate‐purging. Contigs were screened for contamination with BlobToolKit v2.6.5 (Challis et al. 2020), which combines sequence similarity, GC content, and per‐contig coverage to identify non‐target taxa. We retained contigs whose dominant taxon was Arthropoda (with Arthropoda BUSCO hits using the Arthropoda lineage dataset (arthropoda_odb10)), with GC content between 0.30 and 0.45; a window centered on the main GC peak of the *E. texana* assembly (~38%–39% GC), and removed contigs assigned to non‐arthropod taxa outside this range as contaminants; contigs lacking confident taxonomic assignment but falling within this GC interval and the main coverage band were retained as putative *E. texana* sequence. The resulting clean contigs were mapped to ZZ HiFi reads with minimap2 v2.14 (Li 2018) to isolate reads originating from *E. texana* , which were reassembled with the VGP pipeline to produce the final ZZ de novo assembly. Assembly completeness and quality were assessed using BUSCO (Galaxy v5.3.2 + galaxy0 in MetaEuk genome mode; Arthropoda_odb10—created January 8, 2024; 90 reference genomes; 1013 BUSCO groups) (Simão et al. 2015) and QUAST (Galaxy v5.2.0 + galaxy1) (Gurevich et al. 2013).

### 2.7 Reference Genome Curation

As a reference, we used the published scaffold‐level WW hermaphrodite genome assembly of *E. texana* (Bioproject PRJNA352082, accession NKDA00000000; 120 Mb, 108 scaffolds) (Baldwin‐Brown et al. 2018). We re‐evaluated this assembly with BlobToolKit v2.6.5, applying the same contamination‐filtering criteria used for the ZZ assembly to remove non‐target scaffolds and generate a curated WW reference.

### 2.8 Gap‐Filling in the Reference Genome Assembly

We filled gaps in the curated WW reference using ZZ HiFi reads, following the protocol of Volarić et al. (2022), with TGS‐GapCloser v1.2.1 (Xu et al. 2020) with minimap2 v2.14 (Li 2018) for read alignment.

### 2.9 Reference‐Guided Scaffolding

We scaffolded ZZ contigs of the de novo assembly onto the gap‐filled WW reference using RagTag v2.1.0 (Alonge et al. 2022) as in Volarić et al. (2022), producing a scaffold‐level reference‐guided ZZ assembly. RagTag assigns each query contig as an intact unit to a reference scaffold group according to its overall alignment support; it does not subdivide a query contig among reference scaffolds based on local alignment targets. For each query contig, RagTag reports grouping, location, and orientation confidence scores (0–1) calculated from merged reference alignments. Lower location confidence values indicate less decisive placement based on the available alignment evidence and may reflect repetitive sequence, incomplete haplotype resolution or residual heterozygosity in the WW reference, structural variation or genuine ZZ‐WW divergence. ZZ contigs assigned to WW reference scaffolds were concatenated into scaffolded units, with gap sequences inserted between non‐overlapping contigs. RagTag scaffolding did not modify the underlying ZZ contig sequences. The completeness and assembly statistics of the scaffold‐level ZZ assembly were evaluated using BUSCO (Galaxy v5.3.2 + galaxy0 in MetaEuk genome mode; Arthropoda_odb10—created January 8, 2024; 90 reference genomes; 1013 BUSCO groups). Alignments between the WW reference and the scaffold‐level reference‐guided ZZ assembly were visualized using dotPlotly (https://github.com/tpoorten/dotPlotly).

To quantify nucleotide‐level correspondence between corresponding scaffold pairs, we aligned the scaffold‐level ZZ assembly as query against the WW reference as target using minimap2 v2.26 with the asm10 preset and base‐level alignment output (‐c ‐‐cs=long). We retained primary minimap2 alignments (PAF records marked “tp:A:P”) linking each ZZ scaffold to the WW scaffold with the corresponding identifier, excluded cross‐scaffold alignments, and retained alignment blocks ≥ 10 kb. For each PAF record, alignment identity was calculated as the number of matching bases divided by the alignment‐block length. Overlapping intervals were merged on WW target coordinates, and coverage was calculated as the percentage of the WW target scaffold covered by merged, non‐overlapping primary minimap2 alignments from the corresponding ZZ scaffold, filtered at ≥ 80% and ≥ 90% identity. WW scaffold lengths were obtained from the input FASTA. As a sensitivity analysis, we repeated the calculation using all retained minimap2 alignment records, including primary and secondary/supplementary alignments, while retaining the same ≥ 10‐kb, identity‐threshold, and corresponding‐scaffold‐pair filters.

### 2.10 Repeat Analyses

We constructed a de novo repeat library for the ZZ genome with RepeatModeler v2.0.5 (https://www.repeatmasker.org/RepeatModeler/) (default parameters) and masked repeats with RepeatMasker v4.2.1 (https://www.repeatmasker.org/RepeatMasker/) (rmblast engine). RepeatMasker outputs were converted to GFF3 and used as repeat features in the MAKER annotation pipeline.

### 2.11 Genome Annotation

We annotated the repeat‐masked ZZ genome with MAKER (Galaxy version 2.31.11) (Campbell et al. 2014) on the Galaxy platform (https://usegalaxy.org/). As transcript evidence for ZZ males, we assembled RNA‐seq reads from the Baldwin‐Brown et al. (2018) study; where RNA‐seq reads were sequenced from adult males from an outbred natural WAL population, with Trinity v2.15.1 (Haas et al. 2013), which is widely used for de novo transcriptome assembly in non‐model species. Although reference‐guided transcript reconstruction (e.g., mapping reads to the WW genome followed by StringTie) was possible, and both the WW and ZZ assemblies are high‐quality references, we chose de novo assembly with Trinity to derive a ZZ‐specific transcript set without propagating potential annotation biases from the WW genome and to allow recovery of transcripts that might be missing or differently represented in the current WW annotation. The WW genome was previously annotated (Baldwin‐Brown et al. 2018), but we used its annotation only in a comparative context, not as the sole source of transcript evidence, to avoid propagating annotation biases into the ZZ gene set. As protein homology evidence, we used a related branchiopod crustacean ( *Daphnia magna* ) proteome from Uniprot, chosen because most arthropod BUSCO genes identified in the ZZ assembly by BlobToolkit matched *D. magna* and no comparably comprehensive proteome from a more closely related branchiopod species was available. During gene prediction, we supplied MAKER with the repeat‐masked ZZ genome and the corresponding repeat GFF3 file to prevent transcripts from aligning to repetitive elements. After an initial annotation round, we evaluated completeness with standard annotation statistics (Galaxy version 0.8.4) and BUSCO (Galaxy version 4.1.4; Arthropoda_odb10), then trained the ab initio gene predictors SNAP (Galaxy version 2013_11_29 + galaxy1) (Korf 2004) and AUGUSTUS (Galaxy Version 3.4.0 + galaxy1) (Stanke and Morgenstern 2005) and reran MAKER for two additional rounds to refine gene models. We chose MAKER because it provides a mature, widely‐used framework that integrates de novo transcript assemblies, protein homology, and multiple ab initio predictors, and is therefore well suited for annotating non‐model genomes. Newer pipelines (e.g., BRAKER, CAT, or NCBI EGAP) could also be applied, but MAKER allowed us to combine our transcript and protein evidence with iterative training of SNAP and AUGUSTUS all within a single, well‐tested workflow.

### 2.12 Scaffold‐Specific Length, Repeat and Gene Content Analysis

To evaluate scaffold‐specific sequence composition, we summarized length, repeat, and gene annotations for each corresponding ZZ and WW scaffold. Scaffold length was calculated from the assembled scaffold sequence. Repeat count was defined as the number of annotated repeat intervals, and repeat length was calculated as the total annotated repeat span per scaffold. Gene count was defined as the number of annotated gene features per scaffold, and total gene span was calculated as the cumulative length of annotated gene intervals. Repeat proportion and gene‐span proportion were calculated by dividing the respective annotated span by total scaffold length. Repeat and gene annotations were evaluated independently; consequently, their spans may overlap and do not sum to total scaffold length.

## 3 Results and Discussion

### 3.1 Sequencing Data

From the male shrimp, the application of the Qiagen Magattract kit with the HMW DNA protocol yielded 529.7 ng of high molecular weight gDNA. This gDNA was then used to construct ultra‐low libraries for subsequent sequencing. The sequencing yielded 1,945,182 HiFi reads, totaling over 17 Gb in length at 99.9% accuracy. The HiFi reads exhibited a maximum length of 32 kb and a minimum length of 163 bp. The bulk of the reads were 4 kb–15 kb with a mean read length of 9 kb. The quality assessment of HiFi reads evaluated using FastQC showed that there were no remaining adapters or specific overrepresented sequences present.

### 3.2 Genome Size Evaluation

According to GenomeScope, the genome size for the raw ZZ HiFi sequencing reads was estimated to be 103 Mb with 88.4% representing unique sequences and the remaining portion being repeats. Moreover, 97.7% of the genome was homozygous with the remaining 2.3% heterozygous and the average coverage of the reads was recorded at 76.5×. *Eulimnadia texana* is naturally inbreeding (Weeks and Zucker 1999) and these males were derived from a line that experienced eight generations of selfing in WW hermaphrodites, followed by a cross with non‐inbred ZZ males and finally allowed to breed for two additional generations (Weeks 2004; Calabrese and Weeks 2018). Given this breeding history, we expect overall nucleotide diversity to be low but not strictly zero. However, the heterozygosity reported by GenomeScope is a *k*‐mer based model estimate rather than a direct genome‐wide SNP rate, and in a repeat‐rich genome it likely reflects a combination of true residual variation and technical factors (e.g., repetitive or difficult‐to‐assemble regions, sequencing artifacts, or low‐level contaminants) rather than high standing nucleotide diversity in this inbred line.

Although the GenomeScope analysis of raw ZZ HiFi sequencing reads was successful (see above), the *k*‐mer profile showed three peaks (Figure 1a), suggesting contamination. After contamination‐filtering with BlobToolKit and rerunning GenomeScope on cleaned reads (see below), the estimated genome size was 87 Mb with 88.2% unique sequence, 97.9% homozygosity, and ~79× coverage, and the *k*‐mer profile reverted to the expected two‐peak pattern (Figure 1b). These GenomeScope estimates (87–103 Mb) are lower than the ~113 Mb sizes of the final de novo ZZ assembly (see below) and the curated WW reference. In a highly homozygous, repeat‐rich genome such as *E. texana* , *k*‐mer modeling at *k* = 21 can underestimate genome size because heterozygous and homozygous peaks are not well separated and repetitive *k*‐mers distort the fit to the expected mixture distribution; short *k*‐mers are particularly sensitive to these effects. We therefore interpret the GenomeScope values as lower‐bound approximations and treat the convergent ~113 Mb sizes of the ZZ and WW assemblies, together with their high Arthropoda BUSCO completeness, as our best estimate of nuclear genome size. These results are consistent with a predominantly homozygous, highly inbred genome, with the remaining heterozygous fraction likely reflecting a mix of residual variation and technical factors rather than prevalent high nucleotide diversity.

**FIGURE 1:**
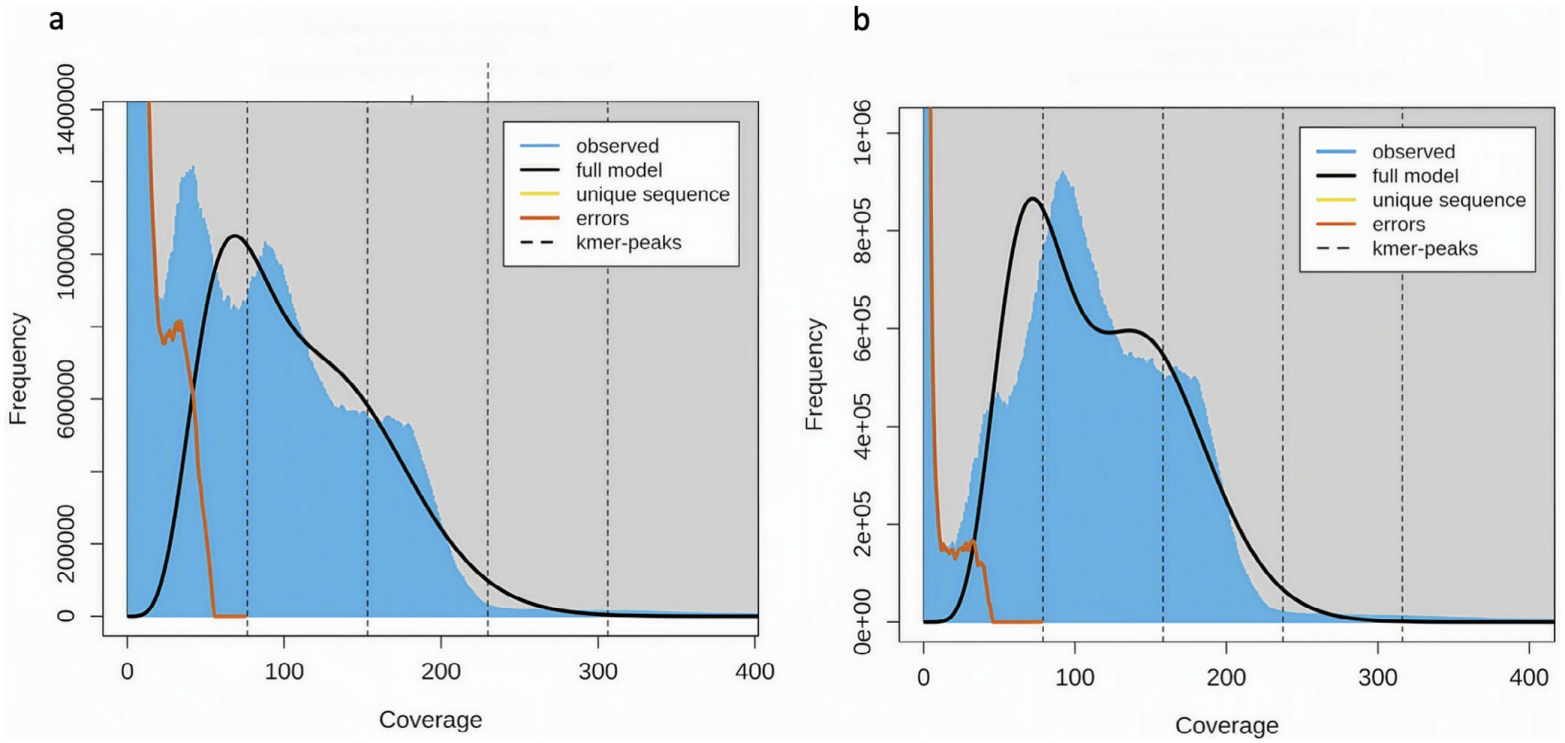
GenomeScope *k*‐mer profiles for *Eulimnadia texana* ZZ HiFi reads before and after contamination filtering. (a) GenomeScope 2.0 profile generated from raw ZZ HiFi reads using 21‐mers. The model estimated a genome size of approximately 103 Mb, 88.4% unique sequence, 97.7% homozygosity, and approximately 76.5× read coverage. The three‐peak *k*‐mer distribution suggested the presence of non‐target sequence. (b) GenomeScope 2.0 profile generated after BlobToolKit‐based contamination filtering and read remapping. The model estimated a genome size of approximately 87 Mb, 88.2% unique sequence, 97.9% homozygosity, and approximately 79× coverage. The filtered reads showed the expected two‐peak distribution. GenomeScope‐derived genome‐size estimates are treated as lower‐bound approximations because *k*‐mer modeling of this highly homozygous, repeat‐rich genome may underestimate assembly span.

### 3.3 De Novo Genome Assembly

HiFiasm produced preliminary primary and alternate contig‐level de novo assemblies of 230 and 49 Mb, respectively (Table S1). Contigs from both assemblies were screened for contamination with BlobToolKit, retaining contigs with Arthropoda hits and GC content between 0.30 and 0.45 as “clean” *E. texana* sequences (Figure 2; Table S1). Clean contigs were used to identify *E. texana* HiFi reads by remapping with minimap2, and these reads were reassembled with the VGP pipeline. After duplicate purging and a second round of BlobToolKit filtering, the final de novo ZZ assembly comprised 97 contigs totaling ~113 Mb. Among these, 27 contigs spanning 110 Mb contained arthropod BUSCO genes. The final contig level de novo assembly had an N50 of 3.5 Mb, a maximum contig length of 10 Mb, and GC content of 38.77% (Table 1; Table S2). BUSCO analysis of the filtered 97 contig ZZ assembly reported 98.1% complete BUSCOs (94.5% single‐copy, 3.6% duplicated, 0.9% fragmented and 1.0% missing; Table 2), indicating a highly complete, contamination‐filtered ZZ genome assembly.

**FIGURE 2:**
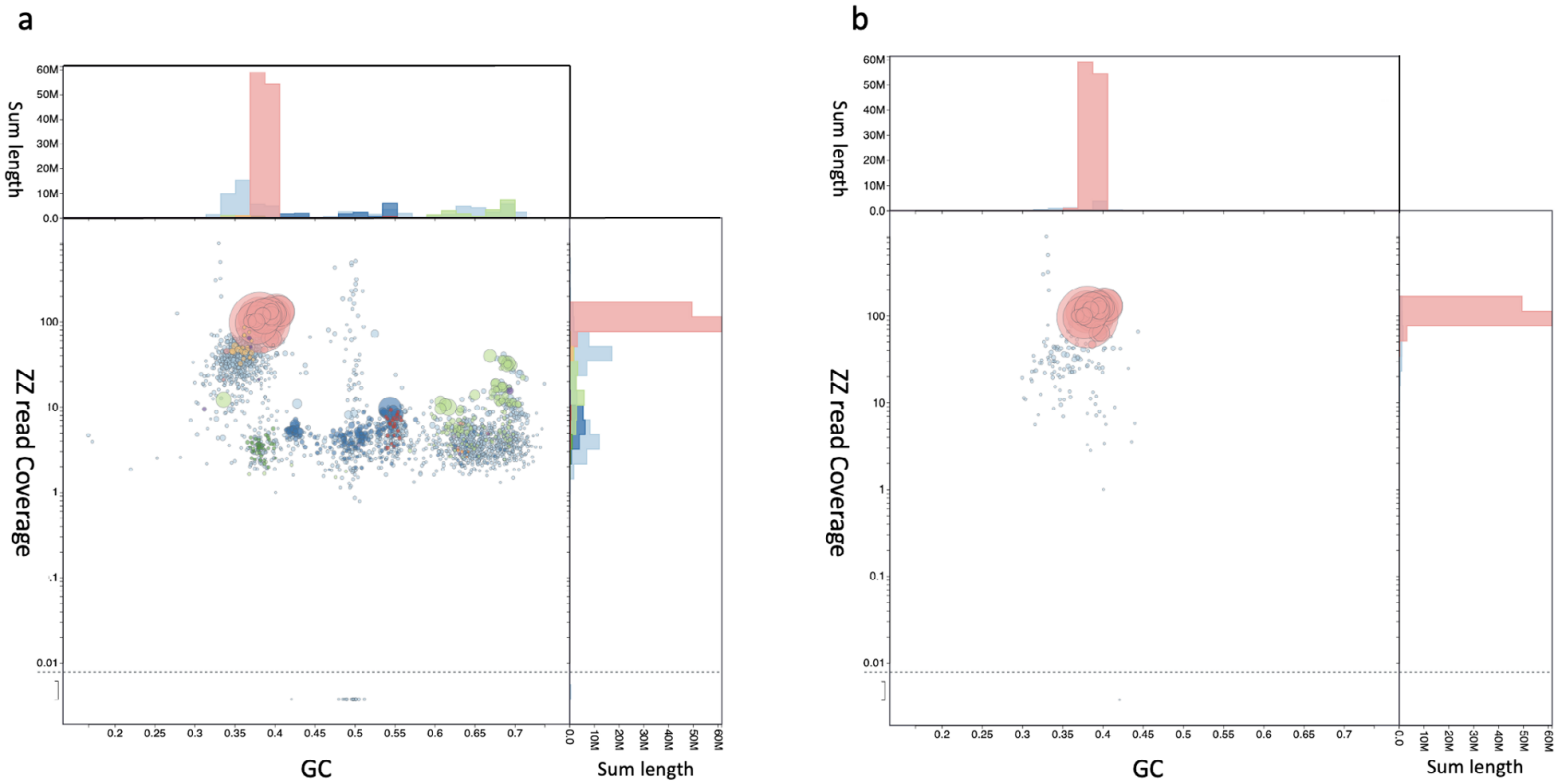
Blob plot of base coverage in raw ZZ HiFi reads (M64108E) against the GC proportion for sequences in the primary ZZ *Eulimnadia texana* assembly. (a) Raw ZZ HiFi reads. (b) Clean ZZ HiFi reads after filtering. Each point represents a contig; point area is proportional to contig length, colors indicate taxonomic assignment, and marginal histograms show the distribution of total assembled sequence along the coverage and GC‐content axes. Phyla designations: total = gray; no‐hit = light blue; Bacteroidota = dark blue; Pseudomonadota = light green; Ascomycota = dark green; Arthropoda = pink; Deinococcota = red; Ciliophora = light orange; Verrucomicrobiota = dark orange; Streptophyta = light purple; other = dark purple.

**TABLE 1:** Comparison of assembly statistics between the de novo ZZ genome assembly and the WW assembly filtered by the BlobToolKit pipeline.

Assembly statistics	Final version of de novo ZZ assembly	Cleaned WW assembly
#Contigs	97	44
Largest Contig	10,087,145	42,684,797
Total length	112,727,327	112,861,194
N50	3,561,963	10,428,323
L50	11	3
GC (%)	38.77	38.78

**TABLE 2:** Genome completeness for the final de novo 97 contig‐level ZZ assembly and 12 scaffold‐level ZZ assembly using the BUSCO analysis (Galaxy v5.3.2 + galaxy0; arthropoda_odb10).

BUSCO genes	97 Contigs, #genes (%)	12 Scaffolds, #genes (%)
Complete (S)	993 (98.1%)	993 (98.1%)
Complete and single copy (S)	957 (94.5%)	957 (94.5%)
Complete and duplicated (D)	36 (3.6%)	36 (3.6%)
Fragmented (F)	9 (0.9%)	9 (0.9%)
Missing (M)	11 (1.0%)	11 (1.0%)
Total BUSCO groups searched	1013 (100%)	1013 (100%)

### 3.4 Reference‐Guided Genome Assembly

Due to its highly contiguous nature, we used the WW hermaphrodite genome assembly of *Eulimnadia texana* as the reference genome (Baldwin‐Brown et al. 2018). The published WW genome assembly was 120 Mb with 108 scaffolds (Figure 3a). However, after contamination filtering with BlobToolKit it was reduced to ~113 Mb in 42 scaffolds (44 contigs) with an N50 of 10 Mb (Table 1). Among these, 14 scaffolds spanning 110 Mb contained arthropod BUSCO genes (Figure 3b), comparable to the 110 Mb of arthropod BUSCO‐containing sequence in the contig level de novo ZZ assembly, indicating similar functional gene content. Consistent with this, the cleaned WW reference and ZZ de novo assembly also showed similar total genome lengths and GC content (Table 1).

**FIGURE 3:**
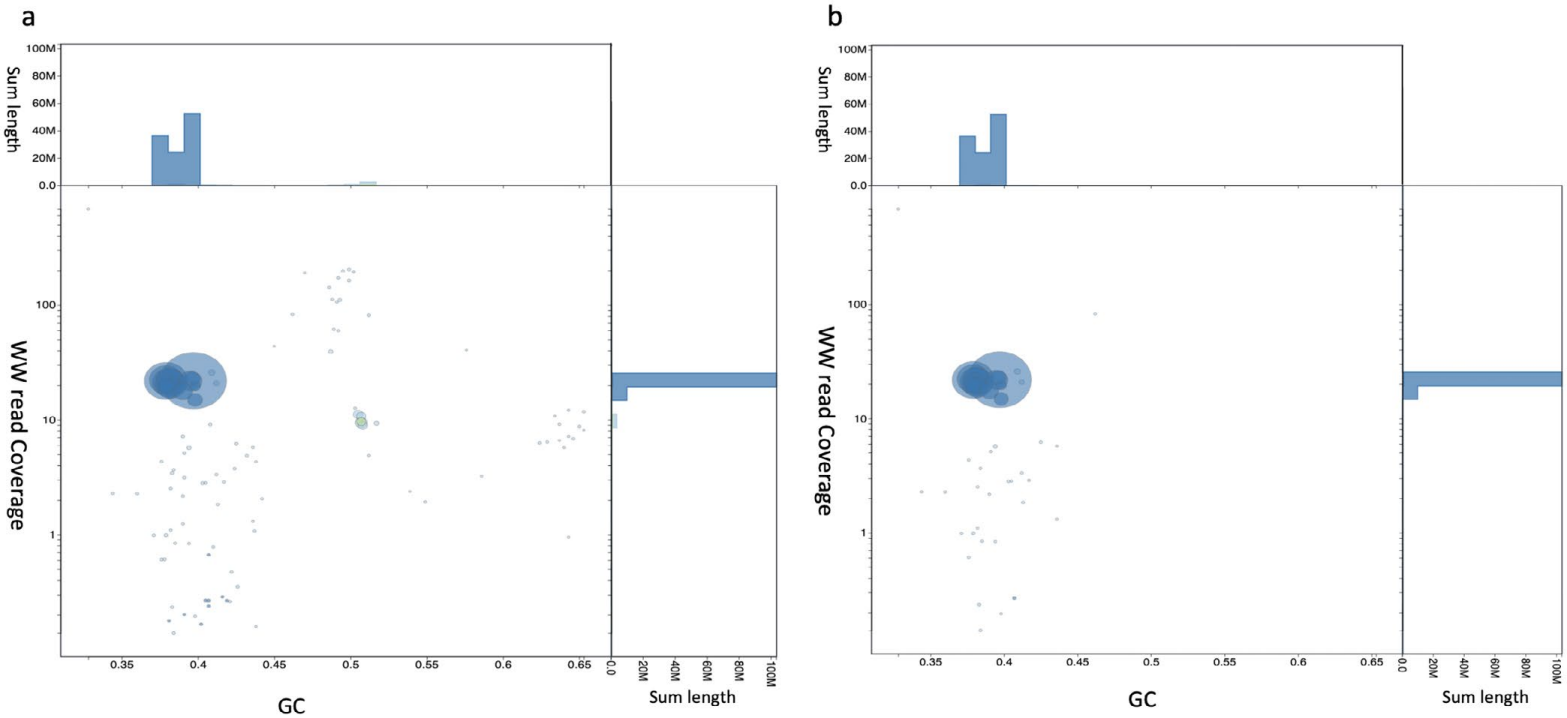
Blob plots of base coverage in the WW reads (SRR47877521) against the GC proportion for sequences in the WW *Eulimnadia texana* assembly. In panel (a), all sequences in the WW assembly are shown; in panel (b), the assembly has been filtered to exclude sequences lacking arthropod BUSCO genes (putative contaminants). Each point represents a contig; point area is proportional to contig length, colors indicate taxonomic assignment, and marginal histograms show the distribution of total assembled sequence along the coverage and GC‐content axes. The phyla designations in the figure are the same as in Figure 2.

For reference‐guided scaffolding, we first filled gaps in the curated WW reference with ZZ HiFi reads using TGS‐GapCloser, then used RagTag to align and orient ZZ contigs from the de novo ZZ assembly onto the gap‐filled reference assembly based on confidence scores (Table S3). RagTag placed 49 of 97 ZZ contigs onto the 11 largest WW scaffolds (Table 3), where 17 of the ZZ contigs mapped to the putative sex chromosome scaffold C0001 (which is ~43 Mb in both ZZ and WW assemblies) (Baldwin‐Brown et al. 2018). Although 48 of 97 contigs were placed in the unlocalized “Chr0_RagTag” scaffold, these were predominantly short and together represented only 1.1% of the ZZ assembly. Thus 98.9% of the assembled ZZ sequence was assigned to the 11 major WW scaffold groups (Table 3). These unplaced contigs were relatively shorter than the placed contigs, suggesting that their fragmented nature was likely due to their highly repetitive DNA content, hindering the formation of longer contigs during the assembly process. During scaffolding, RagTag inserts gap placeholders between adjacent ZZ contigs when they do not overlap or cannot be confidently oriented on the WW scaffolds. These gaps contribute to the total reported scaffold level reference‐guided ZZ assembly length without adding new sequence. The Chr0_RagTag scaffold contains ZZ contigs that RagTag did not place into the 11 major WW scaffold groups under the selected scaffolding criteria, further increasing the apparent size of the finalized scaffold‐level ZZ genome assembly. The scaffolded ZZ genome comprised 12 scaffolds (11 WW scaffolds and Chr0 scaffold) constructed from 97 contigs, with a total length of ~113 Mb and only 0.18% gap sequence. The scaffold N50 was 18 Mb (contig N50 3 Mb), indicating chromosome‐scale contiguity. BUSCO analysis of the 12 scaffold ZZ assembly reported 98.1% complete Arthropoda orthologs (94.5% single‐copy, 3.6% duplicated), 0.9% fragmented, and 1.0% missing (Table 2).

**TABLE 3:** Statistics for scaffolding ZZ contigs onto WW genome scaffolds using RagTag.

WW scaffold	WW scaffold length (bp)	Number of ZZ contigs placed	Cumulative length of ZZ contigs placed (bp)
C0001^a^	42,684,797	17	43,926,626
C0002	18,070,303	9	18,364,870
C0003	10,428,323	5	10,317,972
C0004	10,182,658	4	13,958,433
C0005	6,112,661	4	6,001,896
C0006	5,501,975	1	2,931,060
C0007	4,755,267	2	2,253,342
C0008	4,627,357	2	8,423,200
C0009	3,262,412	1	3,179,627
C0011	2,191,986	3	1,391,044
C0015	746,784	1	774,479
Total placed ZZ contigs		49	111,522,549
		Percentage	98.9%
Chr0 (unplaced ZZ contigs)		48	1,204,778
		Percentage	1.1%
Total ZZ contigs		97	112,727,327
		Percentage	100%

*Note:* ZZ contigs were categorized as “placed” if they mapped to WW scaffolds and “unplaced” (Chr0_RagTag) if they did not. “Cumulative length of ZZ contigs placed” is the sum of full ZZ contig lengths with one or more alignments to each WW scaffold; due to partial alignment of contigs, these cumulative lengths can exceed the scaffold length. Percentages represent the proportions of total ZZ contig length that placed onto WW scaffolds vs. remaining in Chr0 (unplaced), summarizing the fraction of ZZ assembly anchored to the WW scaffold‐level genome.

^a^ The presumed sex chromosome.

To visualize genome‐wide structural similarity between ZZ and WW, we plotted alignments of ZZ contigs to WW scaffolds (Figure 4) and then quantified scaffold‐by‐scaffold coverage (Table 4). The alignment between the WW reference assembly and the scaffold‐level ZZ genome assembly generated by the RagTag algorithm was visualized via a dot plot (Figure 4). Most of the ZZ contigs exhibited continuous alignment with the scaffolds in the WW reference assembly, showcasing an average to high mean percent identity (per query) (Figure 4). Some ZZ contigs show alignments to more than one WW scaffold in Figure 4, reflecting shared or repetitive sequence across scaffolds, but RagTag assigns each contig to a single WW scaffold in the scaffolded ZZ genome.

**FIGURE 4:**
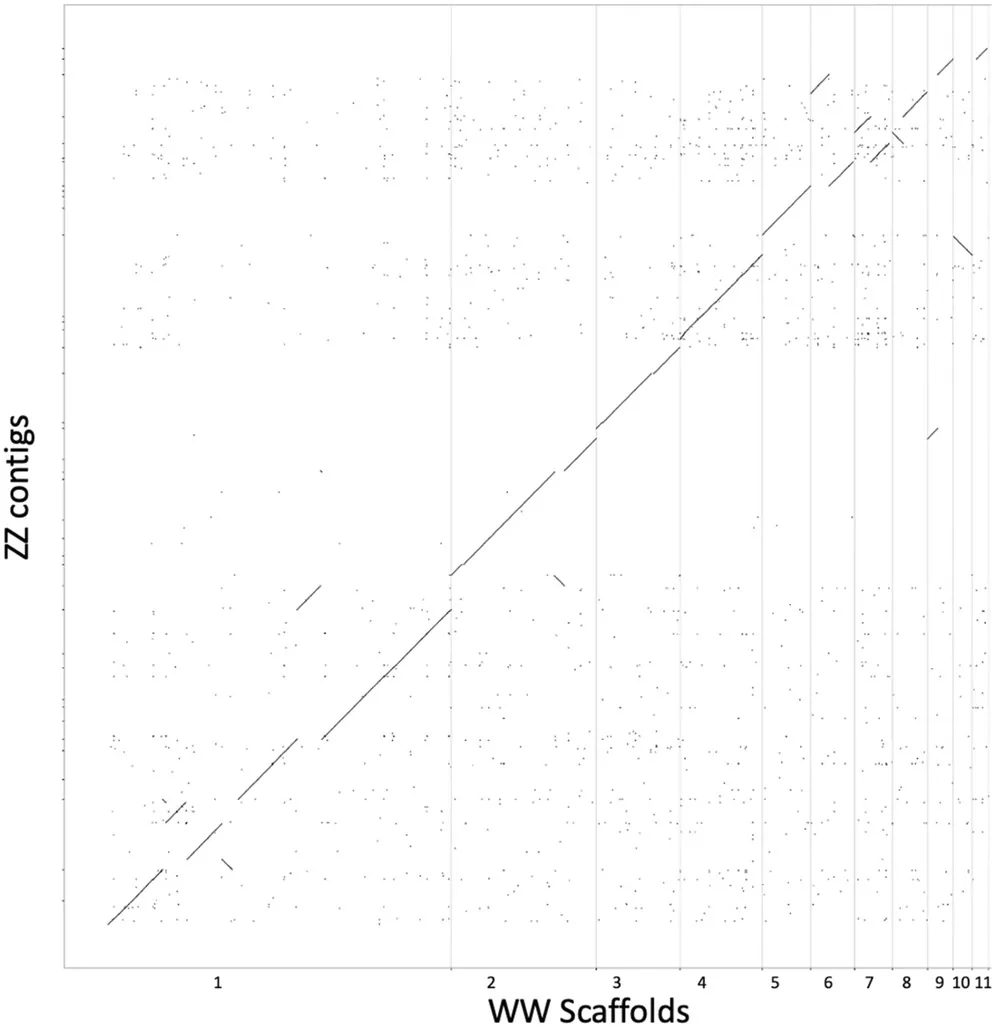
Whole‐genome dot plot comparing the scaffolds of WW reference assembly and the ZZ contigs visualized via dotPlotly. Each line represents an alignment between a ZZ contig and a WW scaffold, so the plot summarizes genome‐wide structural similarity. Autosomal scaffolds (e.g., C0002, C0003, C0005, C0009) show dense, continuous alignments, whereas the putative sex‐chromosome scaffold (C0001) exhibits sparser, fragmented alignments, highlighting that most structural divergence is concentrated on the sex chromosome.

**TABLE 4:** WW scaffold coverage by corresponding ZZ scaffolds in primary ≥ 10‐kb base‐level alignments at ≥ 80% and ≥ 90% identity.

WW scaffold	WW scaffold length (bp)	Covered by ZZ bp (≥ 90% identity)	% Covered by ZZ (≥ 90% identity)	Covered by ZZ bp (≥ 80% identity)	% Covered by ZZ (≥ 80% identity)
C0001^a^	42,684,797	29,208,989	68.43	35,354,176	82.83
C0002	18,070,303	16,746,112	92.67	16,746,112	92.67
C0003	10,428,323	10,336,523	99.12	10,336,523	99.12
C0004	10,182,658	5,028,200	49.38	6,686,422	65.66
C0005	6,112,661	5,998,700	98.14	5,998,700	98.14
C0006	5,501,975	1,697,445	30.85	2,146,201	39.01
C0007	4,755,267	1,102,259	23.18	1,637,940	34.44
C0008	4,627,357	1,955,374	42.26	2,990,621	64.63
C0009	3,262,412	1,593,463	48.84	1,593,463	48.84
C0011	2,191,986	1,334,827	60.90	1,334,827	60.90
C0015	746,784	443,922	59.44	443,922	59.44

^a^ The presumed sex chromosome.

To quantify scaffold‐level sequence correspondence between the reference‐guided ZZ assembly and the scaffold‐level WW reference, we generated base‐level whole‐genome alignments and compared corresponding scaffold pairs, treating the WW assembly as the target/reference and the ZZ assembly as the query. For each scaffold pair, we calculated the proportion of the WW scaffold length covered by merged, non‐overlapping primary ZZ alignments of at least 10 kb that met identity thresholds of ≥ 80% or ≥ 90% (Table 4). These values are measures of high‐identity alignment coverage and do not represent mean nucleotide identity across an entire scaffold or genome. This analysis differs from the 98.9% value reported in Table 3, which represents the proportion of the ZZ assembly contained in contigs assigned by RagTag to WW scaffolds during reference‐guided scaffolding and does not quantify base‐level sequence coverage or nucleotide identity.

High‐identity coverage varied among corresponding scaffold pairs. At the ≥ 90% threshold, the corresponding ZZ scaffolds covered 92.67% of WW C0002, 99.12% of WW C0003, and 98.14% of WW C0005. The putative sex‐chromosome scaffold C0001 was covered by 68.43% at ≥ 90% identity and 82.83% at ≥ 80% identity. Among the other large scaffolds, coverage at ≥ 90% identity was 49.38% for C0004, 30.85% for C0006, 23.18% for C0007, and 42.26% for C0008; coverage at ≥ 80% identity increased to 65.66%, 39.01%, 34.44%, and 64.63%, respectively. Thus, the broad collinearity observed in the ZZ–WW dot plot is accompanied by substantial, but heterogeneous, high‐identity sequence correspondence among the largest scaffold pairs. C0009, C0011, and C0015 showed 48.84%, 60.90%, and 59.44% coverage, respectively, at both ≥ 80% and ≥ 90% identity, indicating that the aligned portions of these scaffolds were highly similar but that only part of each scaffold was represented by retained same‐pair alignments. Inclusion of all PAF alignment classes produced nearly identical scaffold‐coverage estimates, indicating that exclusion of secondary alignments did not materially affect the observed differences among the major scaffold pairs (Table S4). Overall, the dot plot demonstrates broad collinearity of large scaffold groups, whereas Table 4 quantifies local sequence representation under strict same‐pair and identity filters. These measures are therefore complementary; broad macrosynteny can coexist with incomplete coverage by long, high‐identity, corresponding‐scaffold alignment blocks.

### 3.5 Genomic Repetitive Content

Repeat analysis of the ZZ male *E. texana* scaffold‐level genome assembly reported that 34.27 Mb (30.4%) of the genome consisted of regions annotated as repetitive by RepeatModeler and RepeatMasker (Table 5). The majority of these repeats belong to interspersed elements spanning 33.69 Mb (29.89%) of the genome, including rolling‐circles (0.1%), long‐terminal repeats (LTRs, 1.44%), long interspersed nuclear elements (LINEs, 1.49%), short interspersed nuclear elements (SINEs, < 0.01%), DNA transposons (1.97%), and a large fraction of unclassified repeats (24.99%). Because *E. texana* is a non‐model species and our de novo RepeatModeler library has not yet undergone manual curation, these estimates should be viewed as a broad, first‐pass summary based on de novo repeat family discovery and standard masking, rather than a fully resolved TE landscape. A detailed curation of *E. texana* specific TE families (including classification and evolutionary analyses) is in preparation.

**TABLE 5:** Repeat content composition for the ZZ assembly of *Eulimnadia texana* .

	Number of elements	Length (bp)	Percentage
**Retroelements**	8029	3,304,446	2.93%
SINEs	17	724	< 0.01%
Penelope	187	118,034	0.10%
LINEs	4957	1,675,932	1.49%
R1/LOA/Jockey	1215	443,633	0.39%
RTE/Bov‐B	27	9807	0.01%
L1/CIN4	2576	562,883	0.50%
LTR elements	3055	1,627,790	1.44%
BEL/Pao	590	696,840	0.62%
Gypsy/DIRS1	667	513,020	0.46%
**DNA transposons**	7653	2,217,077	1.97%
hobo‐Activator	2123	707,229	0.63%
Other (Mirage, P‐element, Transib)	692	245,000	0.22%
Rolling‐circles	445	114,391	0.1%
Unclassified	133,969	28,171,975	24.99%
Total interspersed repeats		33,693,498	29.89%
Small RNA	778	251,823	0.22%
Satellites	44	15,545	0.01%
Simple repeats	1593	191,142	0.17%

*Note:* No. of sequences: 97; Total length: 112,727,245 bp (excluding N/X‐runs); GC level: 38.77%; Bases masked: 34,266,399 bp (30.40%).

### 3.6 Genome Annotation

We identified 21,165 genes in the scaffold‐level male *E. texana* genome, with an average gene length of 3842 bp, by employing the MAKER pipeline along with ab initio gene predictors (Table S5). The cumulative length of these genes was reported at 81 Mb, constituting 72% of the genome. Among these, 27% was occupied by 128,726 exons spanning 31 Mb, while the remaining 45% was occupied by 107,561 introns spanning 51 Mb of the genome (Table S5). The maximum and mean number of transcripts per gene were 1, with an average transcript size of 1448 bp (Table S6). There were 128,726 distinct exons with a mean of 6.1 distinct exons per gene (Table S6). The majority of the genes were multi‐exon (20,103; 95%), while 1062 (5%) were single‐exon genes (Table S6). The genome annotation had a complete BUSCO score of 95.1%, with 87.2% being single‐copy and 7.9% being duplicated BUSCOs, using an Arthropoda database (Table S6).

Building on our previous work demarcating the Z chromosome (Ediriweera and Weeks 2026) and the gene annotations reported in Ediriweera (2025), we estimate that the WW genome and ZZ genome contain 20,971 and 21,165 genes, respectively, and that the W and Z chromosomes contain 7969 and 8792 genes, respectively (C. Ediriweera and S. C. Weeks, in prep.), indicating broadly similar gene numbers, both genome‐wide and on the proto‐sex chromosomes.

The cumulative gene length (81 Mb; 72% of the 113 Mb genome) includes both exons and introns, and many gene bodies overlap regions annotated as repetitive elements. Since the genome was soft‐masked with RepeatMasker and the repeat GFF3 was supplied to MAKER, repeat‐annotated bases within genes are counted in both the “repeat” and “gene” summaries, so the reported 72% gene coverage and 30.4% repeat coverage are not additive and do not imply that > 100% of the genome is occupied; intergenic and non‐coding regions are present but are partly encompassed by repeat and intronic annotations. The scaffold‐level ZZ annotation (21,165 genes) is modestly higher than the 20,971 genes reported for the WW assembly, and BUSCO scores of 95.1% complete with 7.9% duplicated Arthropoda orthologs suggest that while core gene content is largely recovered, some loci are represented by multiple or fragmented models, particularly in repeat‐rich regions. We therefore consider this gene set a high‐coverage first‐pass annotation that likely includes a modest number of redundant or split models, and expect future manual curation and additional transcriptome data to refine total gene counts and reduce apparent duplication.

### 3.7 Scaffold‐Specific Length, Repeat and Gene Content Between ZZ and WW Genomes

To assess whether differences in sequence composition contributed to heterogeneous ZZ–WW high‐identity alignment coverage, we compared scaffold length, repeat annotations, and gene annotations between corresponding ZZ and WW scaffolds (Table 6). Scaffold pairs with near‐complete ≥ 90% corresponding‐scaffold coverage, including C0002, C0003, and C0005, had closely matched lengths between assemblies, with absolute length differences of 0.11–0.29 Mb. Their repeat fractions were also similar between assemblies. In contrast, ZZ C0004 and C0008 were 3.78 and 3.80 Mb longer, respectively, than their WW counterparts, whereas ZZ C0006 and C0007 were 2.57 and 2.50 Mb shorter. C0011 showed a smaller but comparable pattern of contraction, being 0.80 Mb shorter in ZZ.

**TABLE 6:** Scaffold‐specific length, repeat content, and gene count of corresponding ZZ and WW genome assembly scaffold groups.

Scaffold	WW length (Mb)	ZZ length (Mb)	ZZ − WW length (Mb)	WW repeat %	ZZ repeat %	ZZ − WW repeat bp (Mb)	WW gene count	ZZ gene count	ZZ − WW gene count
C0001	42.68	43.93	+1.24	29.39	28.68	+0.05	7969	8792	+823
C0002	18.07	18.36	+0.29	39.08	38.03	−0.08	3134	3504	+370
C0003	10.43	10.32	−0.11	40.45	39.40	−0.15	1678	1850	+172
C0004	10.18	13.96	+3.78	44.30	43.43	+1.55	1474	1661	+187
C0005	6.11	6.00	−0.11	43.44	42.18	−0.12	968	1043	+75
C0006	5.50	2.93	−2.57	40.74	39.17	−1.09	953	597	−356
C0007	4.76	2.25	−2.50	41.10	33.26	−1.20	835	356	−479
C0008	4.63	8.42	+3.80	42.14	41.57	+1.55	814	906	+92
C0009	3.26	3.18	−0.08	36.54	31.41	−0.19	657	728	+71
C0011	2.19	1.39	−0.80	29.13	32.06	−0.19	585	259	−326
C0015	0.75	0.77	+0.03	38.63	44.45	+0.06	133	116	−17

*Note:* Scaffold correspondence follows reference‐guided RagTag assignment of ZZ contigs to WW scaffold groups. Repeat percentage is the proportion of each scaffold represented by annotated repeat intervals. The repeat‐bp difference is calculated as ZZ repeat span minus WW repeat span. Gene count is the number of annotated gene features. Positive values indicate a larger ZZ value; negative values indicate a smaller ZZ value.

Length discordance was accompanied by substantial differences in absolute repeat‐ and gene‐associated sequence. Relative to WW, ZZ C0004 and C0008 each contained approximately 1.55 Mb more repeat‐annotated sequence and contained 187 and 92 additional annotated gene features, respectively. Conversely, ZZ C0006 and C0007 contained 1.09 and 1.20 Mb fewer repeat‐annotated bases and had 356 and 479 fewer annotated genes, respectively. ZZ C0011 also had 326 fewer annotated genes than WW C0011. By contrast, C0001 was 1.24 Mb longer in ZZ but differed by only approximately 0.05 Mb in repeat‐annotated sequence, indicating that scaffold‐length differences cannot uniformly be attributed to repeat expansion or contraction. Repeat proportion alone did not explain high‐identity alignment coverage. C0002, C0003, and C0005 had repeat fractions of 38%–43%, overlapping the repeat fractions of C0004, C0006, and C0008, despite near‐complete ≥ 90% coverage for the former scaffold pairs. C0009, C0011, and C0015 had relatively small length differences but still differed in repeat fraction and gene number, further indicating that repeat fraction or gene count alone does not predict scaffold‐pair correspondence. Instead, the combined data indicate unequal representation of repeat‐associated, gene‐associated, and other sequence among the corresponding assembly scaffold groups.

This pattern should also be interpreted in the context of reference‐guided ZZ scaffolding. RagTag assigns the whole ZZ contig to a single WW scaffold group based on predominant overall alignment support, rather than splitting the ZZ contig into fragments based on local alignments and assigning to multiple WW scaffolds. Therefore, a ZZ contig assigned to C0004 or C0008 may retain segments with local alignments to other WW scaffold groups. In the present comparison, ZZ C0004 contained contig‐derived sequence with local alignments to WW C0006 and C0010, and ZZ C0008 contained contig‐derived sequence with local alignments to WW C0006 and C0007. The marked expansion of ZZ C0004 and C0008, together with contraction of ZZ C0006 and C0007, is therefore consistent with differential allocation of sequence among the reference‐guided scaffold groups. These cross‐scaffold alignments indicate non‐exclusive correspondence among scaffold groups, but do not alone establish the original genomic location of the aligned sequence. Because the high‐identity coverage analysis (Table 4) retained only corresponding ZZ–WW scaffold‐pair alignments and excluded cross‐scaffold alignments, local matches between, for example, ZZ C0004 and WW C0006 were not counted toward either C0004–C0004 or C0006–C0006 same‐pair coverage. Thus, contig‐level assignment, together with unequal repeat‐ and gene‐associated sequence representation, likely contributes to the reduced corresponding‐scaffold coverage observed for C0004, C0006, C0007, and C0008. Local elevations in residual heterozygosity reported previously for WW C0004 and smaller WW scaffolds (Baldwin‐Brown et al. 2018) provide additional context for possible sequence complexity in these regions, but do not quantitatively explain the observed threshold‐based coverage differences.

## 4 Conclusion

In conclusion, the successful assembly and annotation of the ZZ male *Eulimnadia texana* clam shrimp genome offer preliminary insights into the evolutionary development of proto‐sex chromosomes. The claimed proto‐sex chromosomes (Z and W) in clam shrimp were anticipated to exhibit similar functional gene content and physical structure with a minimum level of differentiation. Our results reflect that the ZZ male genome has a comparable size to that of the WW hermaphrodite genome and that both assemblies recover a similar complement of conserved single‐copy BUSCO genes, suggesting broadly similar overall functional gene content and autosomal structure, while whole‐genome alignments reveal substantial divergence concentrated on the sex‐chromosome scaffold and several smaller scaffolds. Consistent with the extensive inbreeding and limited outcrossing in our breeding design, GenomeScope estimated only low levels of heterozygosity (~2%–3%), which we consider as upper bounds on nucleotide diversity in a predominantly homozygous, highly inbred genome. Whole‐genome alignments show that most autosomal WW scaffolds are almost completely covered by high‐identity ZZ sequence, whereas the putative sex‐chromosome scaffold and several small scaffolds have much lower coverage, indicating that most structural divergence is confined to these regions. Gene annotation likewise indicates similar gene numbers genome‐wide and on the sex chromosomes themselves, consistent with proto‐sex chromosomes that have diverged in structure and repeat content but have not yet undergone extensive gene loss. Together with the WW hermaphrodite genome assembly, this ZZ genome assembly provides a robust framework for mapping diverse sequences (e.g., genomic, transcriptomic), facilitating the comprehensive characterization of genomic diversity and gene expression differences between Z and W sex chromosomes. Hence, this ZZ genome assembly not only offers a crucial resource but also allows future studies of sex chromosome evolution of clam shrimps. Building on the paired WW and ZZ annotations, we are currently undertaking follow‐up analyses to identify genes that are specific to, or enriched on, the Z and W chromosomes and to investigate their potential roles in sex determination and sexual differentiation in *E. texana* . We anticipate that this genome assembly will advance genetic research on the biology and evolution of clam shrimp, specifically, and sex chromosome evolution more generally.

## Author Contributions

**Chathumadavi Ediriweera:** conceptualization (lead), data curation (lead), formal analysis (lead), methodology (lead), writing – original draft (lead), writing – review and editing (equal). **Stephen C. Weeks:** conceptualization (supporting), supervision (supporting), writing – original draft (supporting), writing – review and editing (equal).

## Funding

This work was supported by Sigma Xia (G20201001116561958) and by the Tiered Mentoring Program at the University of Akron.

## Conflicts of Interest

The authors declare no conflicts of interest.

## Supporting information


**Table S1:** Preliminary ZZ contig results from before and after contaminant removal used for the VGP pipeline.
**Table S2:** Assembly statistics generated by Quast for the primary and alternate ZZ assemblies from before and after purging duplicates.
**Table S3:** Reference‐guided assignment, ordering and orientation of ZZ contigs onto WW scaffold groups by RagTag. ZZ de novo contigs were aligned to the WW reference and were assigned, ordered and oriented by RagTag to construct the corresponding ZZ scaffolds. The listed WW reference scaffold indicates the scaffold group to which RagTag assigned each ZZ contig. Grouping confidence quantifies support for assigning a contig to a WW reference scaffold group; location confidence quantifies support for its position within the group; orientation confidence quantifies support for strand assignment. Scores range from 0 to 1, with lower confidence indicating more ambiguous assignment, placement, or orientation during reference‐guided scaffolding. Contigs assigned to the unplaced Chr0_RagTag bin were excluded.
**Table S4:** Sensitivity of corresponding ZZ–WW scaffold‐pair coverage to alignment identity threshold and inclusion of non‐primary PAF records. Differences represent all‐alignment coverage minus primary‐only coverage, in percentage points.
**Table S5:** Genomic analysis of exon and intron composition in *Eulimnadia texana* ZZ genes.
**Table S6:** Genome annotation summary for ZZ *Eulimnadia texana* . Mean gene locus size was calculated using the cumulative lengths of first to last exons. Mean transcript size was calculated using the lengths of UTRs and CDSs. BUSCO completeness scores for genome annotation were calculated using the Arthropoda lineage database, arthropoda_odb10. Values enclosed in parentheses are expressed as a percentage of total genes. BUSCO annotation results were categorized into C = complete, S = single‐copy, D = duplicated, F = fragmented or M = missing BUSCOs.

## Data Availability

This Whole Genome Shotgun project has been deposited at DDBJ/ENA/GenBank under the accession JBREAQ000000000. The version described in this paper is version JBREAQ010000000.
