# Supplementary material for: High‐Quality Annotated ZZ Male Genome Assembly of Eulimnadia texana
Source: Ecol Evol. 2026 Oct 23;16(10):e74547. doi: 10.1002/ece3.74547 (PMC13694257; doi:10.1002/ece3.74547)
Supplement: Supplementary file 1 [file ECE3-16-e74547-s001.docx]

Table S1. Preliminary ZZ contig results from before and after contaminant removal used for the VGP pipeline.

| Assemblies | Before Contaminant removal | | After Contaminant removal | |
| --- | --- | --- | --- | --- |
|  | #Contigs | Size (Mb) | #Contigs | Size (Mb) |
| Primary | 2,497 | 230 | 166 | 120 |
| Alternate | 1,652 | 49 | 1,014 | 36 |

Table S2. Assembly statistics generated by Quast for the primary and alternate ZZ assemblies from before and after purging duplicates.

| Assembly Statistics | Before Purging | | After Purging | |
| --- | --- | --- | --- | --- |
|  | Primary assembly | Alternate assembly | Primary assembly | Alternate assembly |
| Largest Contig | 10,087,145 | 956,423 | 10,087,145 | 992,843 |
| Total length | 125,828,813 | 35,187,610 | 117,045,728 | 34,790,251 |
| N50 | 3,254,774 | 124,461 | 3,561,963 | 214,297 |
| L50 | 13 | 64 | 11 | 39 |
| GC (%) | 38.93 | 38.02 | 38.68 | 38.2 |

Table S3. Reference-guided assignment, ordering and orientation of ZZ contigs onto WW scaffold groups by RagTag. ZZ *de novo* contigs were aligned to the WW reference and were assigned, ordered and oriented by RagTag to construct the corresponding ZZ scaffolds. The listed WW reference scaffold indicates the scaffold group to which RagTag assigned each ZZ contig. Grouping confidence quantifies support for assigning a contig to a WW reference scaffold group; location confidence quantifies support for its position within the group; orientation confidence quantifies support for strand assignment. Scores range from 0 to 1, with lower confidence indicating more ambiguous assignment, placement, or orientation during reference-guided scaffolding. Contigs assigned to the unplaced Chr0_RagTag bin were excluded.

| WW reference scaffold group (ZZ RagTag scaffold) | ZZ Contig (Query) | Contig length (bp) | ZZ scaffold coordinates | orientation | Grouping confidence | Location confidence | Orientation confidence |
| --- | --- | --- | --- | --- | --- | --- | --- |
| C0001  C0001  C0001  C0001  C0001  C0001  C0001  C0001  C0001  C0001  C0001  C0001  C0001  C0001  C0001  C0001  C0001  C0002  C0002  C0002  C0002  C0002  C0002  C0002  C0002  C0002  C0003  C0003  C0003  C0003  C0003  C0004  C0004  C0004  C0004  C0005  C0005  C0005  C0005  C0006  C0007  C0007  C0008  C0008  C0009  C0011  C0011  C0011  C0015 | ptg000009l_1  ptg000093l_1  ptg000191l_1  ptg000015l_1  ptg000092l_1  ptg000194l_1  ptg000137l_1  ptg000028l_1  ptg000138l_1  ptg000156l_1  ptg000147l_1  ptg000146l_1  ptg000145l_1  ptg000162l_1  ptg000075l_1  ptg000073l_1  ptg000032l_1  ptg000150l_1  ptg000151l_1  ptg000152l_1  ptg000153l_1  ptg000144l_1  ptg000068l_1  ptg000165l_1  ptg000148l_1  ptg000163l_1  ptg000193l_1  ptg000192l_1  ptg000030l_1  ptg000096l_1  ptg000095l_1  ptg000010l_1  ptg000098l_1  ptg000188l_1  ptg000005l_1  ptg000049l_1  ptg000086l_1  ptg000185l_1  ptg000186l_1  ptg000007l_1  ptg000139l_1  ptg000012l_1  ptg000004l_1  ptg000006l_1  ptg000024l_1  ptg000190l_1  ptg000197l_1  ptg000184l_1  ptg000161l_1 | 2,927,114  3,825,175  283,774  8,650,885  428,582  340,838  2,422,910  3,561,963  1,381,627  4,163,443  32,838  2,200,217  1,755,916  893,945  3,904,289  1,750,083  5,403,017  1,394,580  254,603  1,066,579  2,153,063  2,200,472  5,028,375  911,975  1,599,422  3,755,797  683,787  99,010  6,050,254  303,775  3,181,142  2,292,358  993,964  584,964  10,087,145  3,254,774  1,445,835  651,532  649,751  2,931,058  1,805,309  448,029  3,274,784  5,148,416  3,179,627  56,326  40,122  1,294,596  774,479 | 1-2,927,114  2,978,409-6,803,583  6,803,684–7,087,457  7,087,558-15,738,442  15,738,543–16,167,124  16,168,074–16,508,911  16,512,031–18,934,940  18,941,950–22,503,912  22,504,013–23,885,639  23,885,740–28,049,182  28,049,283–28,082,120  28,082,221–30,282,437  30,283,799–32,039,714  32,041,641–32,935,585  32,935,686–36,839,974  36,909,586–38,659,668  38,659,769–44,062,785  1–1,394,580  1,394,681–1,649,283  1,649,384–2,715,962  2,716,063–4,869,125  4,869,226–7,069,697  7,069,798–12,098,172  12,098,273–13,010,247  13,010,348–14,609,769  14,609,870–18,365,666  1–683,787  686,569–785,578  785,679–6,835,932  6,841,680–7,145,454  7,150,313–10,331,454  1–2,292,358  2,292,459–3,286,422  3,286,523–3,871,486  3,871,587–13,958,731  1–3,254,774  3,260,452–4,706,286  4,706,387–5,357,918  5,358,019–6,007,769  1–2,931,058  1–1,805,309  1,847,949–2,295,977  1–3,274,784  3,274,885–8,423,300  1–3,179,627  1–56,326  56,427–96,548  96,649–1,391,244  1–774,479 | -  +  -  +  +  +  +  -  +  -  -  -  -  +  -  +  -  +  +  +  +  +  -  +  +  +  -  -  +  -  -  -  -  +  +  +  +  +  +  +  +  -  -  -  -  -  +  -  - | 0.997  0.996  0.860  0.996  0.997  1  1  0.994  1  0.707  1  0.999  1  0.999  0.999  1  0.995  1  1  1  1  1  1  1  0.909  0.663  1  1  1  1  1  0.526  1  1  0.740  1  1  1  1  0.999  0.941  0.987  0.527  0.512  0.600  1  1  1  0.552 | 0.0679  0.0718  1  0.193  1  1  0.940  0.964  1  0.998  1  0.861  0.999  1  0.105  1  0.102  1  1  1  1  1  0.996  1  1  1  1  1  1  1  1  0.058  0.969  0.115  0.618  1  1  1  1  0.573  0.143  0.982  0.873  0.831  1  1  1  1  1 | 0.998  0.973  1  0.793  1  1  1  1  1  1  1  1  1  1  1  1  1  1  1  1  1  1  1  1  1  1  1  1  1  1  1  0.997  1  0.992  0.998  1  1  1  1  1  0.984  0.956  1  0.999  1  1  1  1  1 |

Table S4. Sensitivity of corresponding ZZ–WW scaffold-pair coverage to alignment identity threshold and inclusion of non-primary PAF records. Differences represent all-alignment coverage minus primary-only coverage, in percentage points.

| WW scaffold | WW length (bp) | Coverage >=80%, primary (%) | Coverage >=80%, all (%) | Difference | Coverage  >=90%, primary (%) | Coverage >=90%, all (%) | Difference |
| --- | --- | --- | --- | --- | --- | --- | --- |
| C0001 | 42,684,797 | 82.83 | 82.83 | 0.00 | 68.43 | 68.43 | 0 |
| C0002 | 18,070,303 | 92.67 | 92.67 | 0.00 | 92.67 | 92.67 | 0 |
| C0003 | 10,428,323 | 99.12 | 99.12 | 0.00 | 99.12 | 99.12 | 0 |
| C0004 | 10,182,658 | 65.66 | 65.66 | 0.00 | 49.38 | 49.38 | 0 |
| C0005 | 6,112,661 | 98.14 | 98.14 | 0.00 | 98.14 | 98.14 | 0 |
| C0006 | 5,501,975 | 39.01 | 39.01 | 0.00 | 30.85 | 30.85 | 0 |
| C0007 | 4,755,267 | 34.44 | 34.44 | 0.00 | 23.18 | 23.18 | 0 |
| C0008 | 4,627,357 | 64.63 | 64.91 | 0.28 | 42.26 | 42.26 | 0 |
| C0009 | 3,262,412 | 48.84 | 48.84 | 0.00 | 48.84 | 48.84 | 0 |
| C0011 | 2,191,986 | 60.90 | 60.90 | 0.00 | 60.90 | 60.90 | 0 |
| C0015 | 746,784 | 59.44 | 59.44 | 0.00 | 59.44 | 59.44 | 0 |

Table S5. Genomic analysis of exon and intron composition in *Eulimnadia texana* ZZ genes.

|  | %GC | % of genome | Average size | Median size | Number | Total length (Mb) |
| --- | --- | --- | --- | --- | --- | --- |
| Gene  Exon  Intron | 39%  48%  34% | 72%  27%  45% | 3,842  238  471 | 2,177  156  112 | 21,165  128,726  107,561 | 81Mb  31Mb  51Mb |

Table S6. Genome annotation summary for ZZ *Eulimnadia texana*. Mean gene locus size was calculated using the cumulative lengths of first to last exons. Mean transcript size was calculated using the lengths of UTRs and CDSs. BUSCO completeness scores for genome annotation were calculated using the Arthropoda lineage database, arthropoda_odb10. Values enclosed in parentheses are expressed as a percentage of total genes. BUSCO annotation results were categorized into C=complete, S=single-copy, D=duplicated, F=fragmented or M=missing BUSCOs.

Max number of transcripts per gene

Mean exon size

Mean gene locus size (first to last exon)

Mean number of distinct exons per gene

Mean number of transcripts per gene

Mean transcript size (UTR, CDS)

Number of distinct exons

Number of genes

Number of genes with alternative transcript variants

Number of multi-exon genes

Number of predicted transcripts

Number of single-exon genes

BUSCO annotation (arthropoda_odb10)

1

238.1

3842.3

6.1

1

1448

128726

21165

0 (0.00%)

20103 (95.0%)

21165

1062 (5.0%)

C:95.1%(S:87.2%,D:7.9%),F:2.4%,M:2.5%
